# Clinical Molecular Immunohistochemistry Mismatch Repair Mutations in Lynch Syndrome in Patients Under 50 Years: A Systematic Review

**DOI:** 10.3390/biomedicines13051062

**Published:** 2025-04-27

**Authors:** Bogdan Adrian Manta, Adrian Cosmin Ilie, Felicia Marc, Daciana Nistor, Patricia Octavia Mazilu, Claudia Borza

**Affiliations:** 1 Division of Clinical Practical Skills, Faculty of Medicine, “Victor Babes” University of Medicine and Pharmacy Timisoara, 300041 Timisoara, Romania; manta.bogdan@umft.ro; 2 Department III Functional Sciences, Division of Public Health and Management, “Victor Babes” University of Medicine and Pharmacy Timisoara, 300041 Timisoara, Romania; ilie.adrian@umft.ro; 3 Department of Medical Sciences, Faculty of Medicine and Pharmacy, University of Oradea, 410073 Oradea, Romania; 4 Department of Functional Sciences, Physiology, Centre of Imuno-Physiology and Biotechnologies (CIFBIOTEH), “Victor Babes” University of Medicine and Pharmacy Timisoara, Eftimie Murgu Square 2, 300041 Timisoara, Romania; 5 Faculty of Medicine, “Victor Babes” University of Medicine and Pharmacy Timisoara, Eftimie Murgu Square 2, 300041 Timisoara, Romania; patrimazilu@yahoo.com; 6 Department of Functional Sciences, Discipline of Pathophysiology, “Victor Babes” University of Medicine and Pharmacy Timisoara, Eftimie Murgu Square 2, 300041 Timisoara, Romania; borza.claudia@umft.ro; 7 Centre for Translational Research and Systems Medicine, “Victor Babes” University of Medicine and Pharmacy Timisoara, Eftimie Murgu Square 2, 300041 Timisoara, Romania; 8 Centre of Cognitive Research in Pathological Neuro-Psychiatry NEUROPSY-COG, “Victor Babes” University of Medicine and Pharmacy Timisoara, Eftimie Murgu Square 2, 300041 Timisoara, Romania

**Keywords:** Lynch syndrome, mismatch repair deficiency, immunohistochemistry, early-onset colorectal cancer, molecular testing

## Abstract

**Background and Objectives**: Lynch syndrome (LS), an autosomal dominant condition arising from germline mutations in mismatch repair (MMR) genes, is a major cause of hereditary early-onset colorectal cancer (CRC). Although patients diagnosed before age 50 represent a critical subgroup where Lynch syndrome might be more prevalent, data on the precise frequency, clinical outcomes, and molecular correlates remain heterogeneous across studies. This systematic review was conducted to (1) estimate the prevalence of MMR deficiency (dMMR) and confirmed LS in patients diagnosed with CRC before the age of 50, and (2) examine immunohistochemistry (IHC) mismatch repair testing patterns and associated molecular findings (BRAF mutations, MLH1 promoter hypermethylation, somatic MMR gene alterations). **Methods**: Following a predefined search strategy in PubMed, Scopus, and Web of Science, five relevant studies were identified (*n* = 5). Each study comprised patients younger than 50 who underwent IHC-based tumor screening. Data extraction covered demographic details, number of patients tested, proportion with abnormal IHC, frequency of somatic or germline MMR gene mutations, and method of classification into sporadic dMMR vs. LS. Quality assessment was performed using recommended scales for observational studies. **Results**: Among 5 studies totaling 960 early-onset CRC patients, the frequency of dMMR CRC ranged from 8.4% to 19.1%. The confirmed prevalence of LS among all young-onset CRC was between 5.0% and 5.9% in three studies but reached 8.9% in another and 5.1% in yet another. Across all studies, the presence of right-sided tumors and histopathological features such as tumor-infiltrating lymphocytes were more common in dMMR cancers. Incorporation of MLH1-promoter hypermethylation and/or BRAF V600E mutation testing aided discrimination of sporadic dMMR CRC from germline LS cases. **Conclusions**: The prevalence of LS in CRC patients younger than 50 is clinically significant, at approximately 5–9%. Routine IHC-based MMR screening is both feasible and effective for detecting LS in early-onset CRC. Further research is needed to standardize universal testing protocols, delineate the role of additional molecular assays, and ensure comprehensive genetic counseling for at-risk individuals.

## 1. Introduction

Early-onset colorectal cancer (CRC), typically defined as diagnosis before age 50, has generated growing interest because of rising incidence in younger cohorts and the possibility of hereditary syndromes such as Lynch syndrome (LS) [1,2]. Lynch syndrome, also referred to as hereditary nonpolyposis colorectal cancer (HNPCC), is an autosomal dominant disorder caused by germline mutations in DNA mismatch repair (MMR) genes, most frequently *MLH1* and *MSH2*, but also *MSH6* and *PMS2* [3,4]. Loss of MMR function leads to microsatellite instability (MSI) and unique pathological features in the associated tumors [5]. Although LS accounts for an estimated 1% to 3% of all CRC cases overall, the proportion in early-onset CRC populations is considerably higher [6]. Given the significant lifetime cancer risk for LS carriers—including colorectal, endometrial, gastric, ovarian, and other malignancies—timely identification of affected individuals is paramount [7]. Studies have shown that universal tumor testing or targeted testing of younger patients using immunohistochemistry (IHC) or MSI testing can improve LS detection rates [8]. However, the precise yield of these approaches, especially in the under-50 CRC cohort, varies by region and testing protocols [9].

An important factor complicating LS identification is that many patients with a germline MMR mutation do not present with a strong family history of cancer [10]. Family structures are smaller in modern times, and incomplete penetrance can obscure the pattern of inheritance [11]. As a result, reliance on clinical criteria such as the revised Amsterdam or Bethesda guidelines alone can fail to capture a substantial subset of Lynch syndrome carriers [12]. To address this, several groups have called for universal or near-universal screening for MMR deficiency (dMMR) in newly diagnosed CRC cases [13]. Yet, resource constraints or competing clinical demands may limit fully universal protocols in certain settings. As a compromise, focusing on patients younger than 50 years—among whom LS is enriched—is commonly advocated [6]. IHC-based screening for the four key MMR proteins (*MLH1*, *MSH2*, *MSH6*, and *PMS2*) is considered a robust, cost-effective method in these younger CRC patients [14].

Clinically, CRC tumors that are MMR-deficient often display right-sided location, high-grade histology, tumor-infiltrating lymphocytes, and a mucinous or medullary phenotype [15,16]. Moreover, IHC can guide the specific gene likely to be mutated because loss of *MLH1* typically co-occurs with *PMS2* loss, while *MSH2* deficiency often accompanies *MSH6* loss [17]. However, not all dMMR in younger patients indicates LS. Sporadic epigenetic silencing of MLH1—usually confirmed by *MLH1* promoter hypermethylation—and presence of BRAF V600E mutations represent key alternative pathways in a fraction of cases [5]. These sporadic dMMR tumors share some phenotypic similarities with LS tumors but carry distinct clinical management implications, as there is no germline heritable cause [18]. Thus, for a patient exhibiting absent *MLH1*/*PMS2*, reflex *MLH1* hypermethylation testing and/or *BRAF* analysis can help differentiate sporadic from hereditary disease [19].

Referring patients with suspicious IHC findings for definitive germline testing is crucial to confirm or exclude LS [4]. Such genetic workups rely on methods including Sanger sequencing and multiplex ligation-dependent probe amplification (MLPA), although next-generation sequencing (NGS) approaches are increasingly employed [7,8]. When LS is confirmed, the impact on clinical management is profound. Enhanced colonoscopic surveillance can reduce CRC mortality, and prophylactic or earlier interventions may be considered for extracolonic malignancies [9,10]. Moreover, cascade testing among first-degree relatives can identify other at-risk individuals who may benefit from similarly intensive screening [13,14]. Despite these benefits, some patients with abnormal IHC results do not proceed to genetic counseling or testing, often due to logistical or psychosocial barriers [15]. Consequently, a notable proportion of LS cases might remain undiagnosed even when tumor testing reveals dMMR [5].

In recent years, multiple investigators have documented the prevalence of dMMR in younger-onset CRC, reporting figures ranging from ~8% to >20% of these patients, depending on clinical inclusion criteria and local population genetics [16,17]. Of these dMMR tumors, approximately one-half or more may be confirmed as LS upon thorough molecular analysis [18]. Although universal screening programs in Western countries show promise, they are not universally implemented, especially in settings where reimbursement or infrastructure remains limited [20,21]. Therefore, focusing on systematically testing CRC patients under 50 for MMR deficiency remains a practical step in high-volume centers or resource-limited healthcare systems, particularly given the enriched yield of germline mutations in this population [5]. This systematic review aimed to estimate the prevalence of MMR deficiency and confirmed LS in young patients diagnosed with CRC before the age of 50 and examine immunohistochemistry mismatch repair testing patterns and associated molecular findings (BRAF mutations, *MLH1* promoter hypermethylation, somatic MMR gene alterations).

## 2. Materials and Methods

### 2.1. Eligibility Criteria and Research Question

This systematic review aimed to identify studies that specifically examined IHC detection of MMR deficiency in patients younger than 50 diagnosed with CRC. We included original research that provided (1) a consecutive or representative series of early-onset CRC cases (diagnosed at ages < 50), (2) details on the frequency of IHC loss of expression of at least *MLH1* and *MSH2* (preferably including *MSH6* and *PMS2*), (3) data on subsequent germline mutation testing or at least differentiation between sporadic MMR deficiency (e.g., *MLH1* promoter methylation) and true Lynch syndrome (LS) with germline mutation, and (4) numerical outcomes regarding the proportion of confirmed LS. We excluded studies primarily focusing on pediatric CRC (younger than age 18) or those investigating only MSI without immunohistochemistry. Additionally, reviews or meta-analyses without unique patient data, purely in vitro or preclinical articles, conference proceedings without full methods or results, and single-patient case reports were excluded.

Our primary research question was “What is the reported prevalence of mismatch repair-deficient (dMMR) colorectal cancers and confirmed LS in patients younger than 50, based on IHC screening approaches?” Secondary questions concerned (i) how often sporadic *MLH1* promoter hypermethylation or a *BRAF V600E* mutation was found among dMMR tumors; (ii) which clinicopathologic features (e.g., tumor sidedness, mucinous histology, tumor-infiltrating lymphocytes) were associated with dMMR; and (iii) the proportion of patients with abnormal IHC who underwent confirmatory germline testing.

This systematic review synthesizes evidence from five pertinent studies that investigated IHC-based tumor testing in patients diagnosed with CRC before age 50. We examine (1) the prevalence of dMMR in these young CRC cohorts, (2) the proportion of patients who eventually received confirmatory germline testing, (3) the fraction diagnosed with LS, and (4) the role of ancillary molecular tests such as *MLH1* promoter hypermethylation or *BRAF V600E* analysis [22]. By comparing these cohorts, we aim to clarify patterns of MMR deficiency, highlight the real-world implementation of screening protocols, and underscore key best practices for prompt genetic evaluation. Ultimately, understanding how different centers achieve early LS detection in this younger CRC subgroup can inform refinements to local protocols, driving better patient outcomes and more comprehensive prevention strategies for familial cancer [23].

### 2.2. Literature Search Strategy

**Figure 1:**
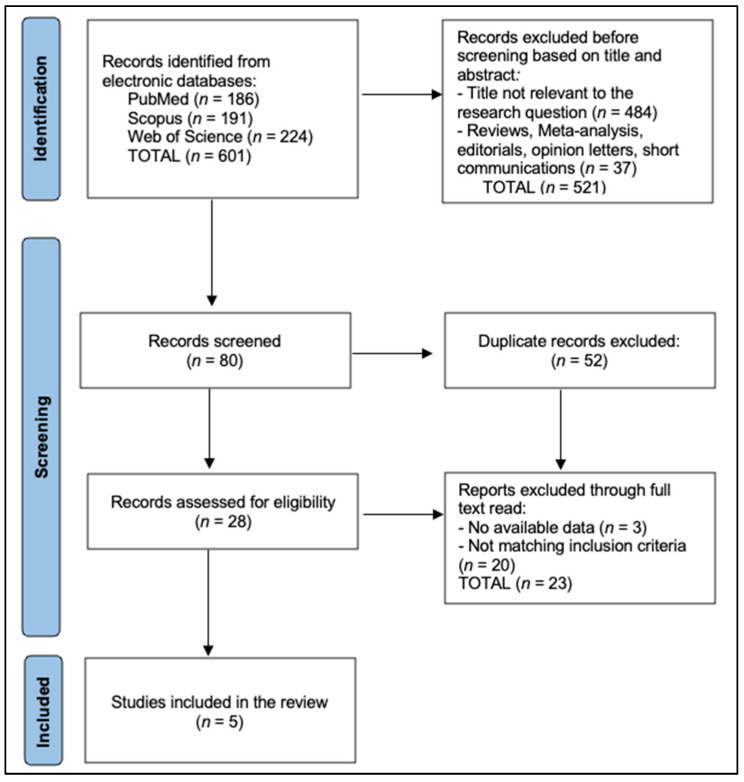
PRISMA flowchart.

The current study followed the PRISMA protocol for systematic reviews and was registered to the Open Science Framework (OSF), with the registration code osf.io/fc7wd. To identify potential articles, we developed a search strategy focusing on early-onset CRC, mismatch repair immunohistochemistry, and Lynch syndrome, as presented in Figure 1. While for the present review we highlight five key studies specifically provided for analysis, we also describe how a typical broad search might be undertaken.

We searched PubMed, EMBASE, and Web of Science using a combination of medical subject headings (MeSH) and free-text terms. Key search terms were “Colorectal Cancer”, “Early-onset”, “Young”, “Immunohistochemistry”, “Mismatch repair deficiency”, “Lynch syndrome”, “Hereditary nonpolyposis colorectal cancer”, “*MLH1*”, “*MSH2*”, “*MSH6*”, and “*PMS2*”. Boolean operators (AND/OR) were used to intersect or combine relevant concepts. The search was restricted to human studies published in English up until December 2024. Two reviewers independently screened the titles and abstracts. Any article suspected of fulfilling the inclusion criteria underwent full-text review. Disagreements regarding inclusion were resolved by consensus or via consultation with a third reviewer (Author C). We also examined reference lists of articles that met inclusion criteria for additional eligible studies.

### 2.3. Data Extraction and Outcomes of Interest

Data extraction concentrated on study design, location and timeframe, total sample size, number of early-onset CRC cases with IHC performed, numbers with lost expression of MMR proteins, the proportion verified to have LS via germline testing, and any additional molecular analyses like *BRAF V600E* or *MLH1* promoter methylation. We also recorded relevant clinical details, including tumor location (right-sided vs. left-sided), presence of multiple synchronous or metachronous lesions, and other histopathological characteristics.

We further collected data on the rate of referral for genetic counseling following abnormal IHC, the fraction of patients who pursued confirmatory genetic testing, and the breakdown of identified germline mutations in *MLH1*, *MSH2*, *MSH6*, and *PMS2*. Where provided, we included age distribution, sex ratio, and family history details. Additionally, we noted if pathologists performed partial IHC panels (e.g., *MLH1* + *MSH2*) or the full four-protein panel that includes *MSH6* and *PMS2*. For each included study, any mention of *MLH1* promoter hypermethylation or *BRAF* mutation testing was noted, especially as it might inform the classification of sporadic versus LS dMMR.

Two reviewers abstracted these data independently; a third reviewer assessed any disagreements. Extracted data were compiled into standardized forms. Finally, we aggregated the main outcomes, focusing on the percentage of early-onset CRC explained by LS, the frequency of sporadic dMMR, and how many patients ultimately underwent confirmatory germline testing. All extracted data were cross-verified to minimize transcription errors.

Because of notable methodological variation (differences in IHC protocols, partial vs. full protein panels, and differential use of molecular confirmatory tests), we opted not to generate a pooled meta-analytic estimate of prevalence. Instead, we performed a descriptive synthesis. Each study’s data on IHC results and the proportion of LS among early-onset CRC were tabulated and compared. We highlight the distribution of MMR proteins lost, the frequency of proven somatic events (e.g., *MLH1* promoter hypermethylation, *BRAF V600E*), and the yield of confirmed germline mutations.

### 2.4. Quality Assessment and Risk of Bias

The included articles were predominantly observational cohorts from single centers or multiple centers. We used a modified Newcastle–Ottawa Scale (NOS) to gauge the methodological quality and risk of bias. Specifically, we assessed the (1) selection of study population, ensuring consecutive or well-defined recruitment of CRC patients <50; (2) ascertainment of exposure (i.e., methods used to confirm LS or dMMR); (3) demonstration that the outcome of interest (LS diagnosis) was not present at the start; (4) adequacy of follow-up, particularly how many abnormal IHC cases proceeded to germline testing; and (5) clarity of reporting regarding confounding variables such as family history or concurrent conditions like ulcerative colitis.

We considered potential biases such as incomplete immunohistochemistry for all four MMR proteins in certain years, or lack of consistent BRAF or methylation testing to exclude sporadic dMMR. Each study was rated independently by two authors for each NOS domain, awarding stars for low risk of bias. An overall rating for each study was produced, and any disagreements were resolved through discussion. Although formal meta-analysis was not performed due to heterogeneity and the small number of studies, evaluating these aspects provided clarity on how representative and reliable each study’s reported prevalence might be.

## 3. Results

Table 1 outlines essential features of each of the five included studies [24,25,26,27,28], illustrating their geographical spread and differences in the methods employed for MMR testing. Notably, the sample sizes of patients under 50 range from as few as 25 in Study 1 [24] to as many as 281 in Study 2 [25]. However, it is important to clarify that Study 1 (Mensenkamp et al. [24]) specifically analyzed 25 “unexplained” MSI-positive tumors without a known germline cause. This narrower focus means that although the study population was smaller, they used additional next-generation sequencing and somatic analyses to identify biallelic events.

Each study utilized IHC as a central screening mechanism, but the actual protocols varied. Study 1 [24] included MSI testing first and then used IHC or extended DNA analysis for selected cases. Studies 2 through 5 primarily used IHC, sometimes in combination with partial MSI or additional markers, to narrow down which tumors might harbor germline mutations [25,26,27,28]. Over time, the availability of robust antibodies for *MSH6* and *PMS2* expanded, so older cohorts, like in Study 2 [25] and part of Study 5 [28], sometimes tested for only *MLH1* and *MSH2*, potentially missing or under-detecting rare isolated *MSH6* or PMS2 deficiency.

All studies mention at least sporadic testing for *BRAF V600E* or *MLH1* promoter hypermethylation in tumors showing *MLH1*/*PMS2* loss, recognizing the significance of these markers in distinguishing sporadic from inherited MMR deficiency. The approach varied, but typically, if a tumor showed *MLH1*/*PMS2* deficiency and either *BRAF* positivity or hypermethylation, it was considered likely sporadic, and germline testing was discouraged or pursued only if clinical suspicion remained high. Finally, the table highlights how germline testing was carried out. Typically, Sanger sequencing was employed, sometimes followed by MLPA for large deletions in *MLH1* or *MSH2*. Studies 1 and 4 mention advanced next-generation sequencing or IonTorrent for certain subsets, reflecting an evolving methodology [24,27].

Table 2 focuses on the core question: among patients diagnosed with CRC before age 50, what fraction exhibit immunohistochemical mismatch repair deficiency (dMMR), and how many are ultimately confirmed to have Lynch syndrome (LS)? Direct prevalence comparisons must be interpreted cautiously because each study applied slightly different inclusion criteria. For instance, Study 1 (Mensenkamp et al. [24]) only analyzed 25 tumors previously identified as MSI-positive but “unexplained” by standard testing. Within that highly selected subset, 13 of 25 (52%) had two somatic mutations. Notably, no germline mutations were identified among them, so the “confirmed LS” was 0%. This perspective is unique, as Study 1 is about elucidating somatic hits in MSI-positive, germline-negative CRC [24].

By contrast, Study 2 (Niessen et al. [25]) had 281 patients younger than 50, finding 25 (8.9%) with dMMR, all of whom turned out to harbor a pathogenic germline mutation. Thus, the fraction of LS among their total <50 population is also 8.9%. Study 3 (Steinhagen et al. [26]) tested 198 early-age CRC patients, showing a higher rate of dMMR (19.1%), but among those, only 10 (5.1% of the total cohort) had confirmed germline mutations. Some of the dMMR cases turned out to be sporadic, typically associated with *MLH1* hypermethylation or *BRAF V600E*, or they had variants of uncertain significance (VUS). Similarly, Study 4 (Suzuki et al. [27]) found 10 patients (8.4%) with dMMR among 119 < 50 CRC cases, of which 7 had LS (5.9%). The remainder had sporadic *MLH1* hypermethylation or inconclusive results.

Finally, Study 5 (Wright et al. [28]) from New Zealand documented 33 (14%) with dMMR among 243 younger-onset CRC. Confirmatory germline testing yielded 12 with LS (5.0% of the entire population). The difference in the proportions of dMMR vs. LS can often be attributed to sporadic epigenetic *MLH1* loss or incomplete test follow-up. Collectively, these data suggest that roughly 8% to 20% of early-onset CRC demonstrates dMMR. However, the final proportion proven to be LS ranges more narrowly between 5% and 9% in large cohorts, highlighting the importance of additional molecular tests to distinguish sporadic from inherited disease.

Table 3 summarizes clinicopathologic findings that were often explored to see if they correlated with mismatch repair deficiency in young-onset CRC. The presence of right-sided (proximal) colonic tumors emerges as a recurring feature. Studies 2, 3, 4, and 5 each reported that a majority (from around 53% up to 80%) of dMMR tumors were located in the right colon, reinforcing earlier observations that Lynch syndrome-associated CRC tends to be right-sided [25,26,27,28].

Another potential histologic marker is the presence of tumor-infiltrating lymphocytes (TILs) or Crohn-like lymphoid reactions. In the setting of dMMR, the accumulation of frameshift peptides can provoke local immunologic responses. Three of these studies reported TILs in more than half of the dMMR cases, although percentages varied. Notably, Study 5’s proportion is somewhat lower (39%), perhaps reflecting differences in pathologist reporting or thresholds for diagnosing a “significant” infiltration [28].

Mucinous or signet-ring differentiation is another recognized feature in dMMR. Studies 3 and 4 reported that up to 50% of dMMR tumors contained mucinous or signet elements, consistent with prior research linking LS and MSI-high tumors to mucinous histology [26,27]. In contrast, Study 2 had a lower rate (20%), suggesting that while mucinous features are important, they are not universal [25].

Finally, the presence of a family history (particularly in a first-degree relative) of early CRC or other Lynch-associated malignancies is historically a central screening criterion. Studies 2, 3, 4, and 5 all indicated moderate to high rates (40–76%) of suspicious family histories among dMMR carriers, but it is noteworthy that a significant fraction of patients with proven LS sometimes lack a robust family history because of small family sizes or incomplete reporting [25,26,27,28].

Table 4 addresses crucial elements of how abnormal immunohistochemistry (IHC) findings translate into confirmatory genetic testing and outcomes [27]. Each study faced the challenge of motivating clinicians and patients to proceed from suspicious IHC results to definitive germline MMR testing. The table reveals that the proportion of patients with abnormal IHC who underwent germline testing varied considerably: 57.8% (Study 3 [26]); 67% (Study 5 [28]); 80% (Study 4 [27]); and 100% in Study 2 [25]. Study 1 [24] used a design that specifically analyzed “unexplained” MSI-positive tumors, effectively a subset that had negative initial germline screening but warranted deeper somatic analysis.

Among those who proceeded to testing, the yield of germline mutations was also variable. In Study 2 [25], all 25 dMMR patients tested were found to have a pathogenic mutation, implying that the entire dMMR subset was indeed LS. In Study 4 [27], 7 of 8 tested had confirmed LS, a rate of 88%. Meanwhile, in Studies 3 and 5, rates of 46% and 45% were found among those tested. Even so, each study highlighted that many abnormal IHC results reflect sporadic dMMR, particularly via *MLH1* promoter hypermethylation or *BRAF V600E* mutation. Studies 3 and 4 detail how these molecular analyses can reclassify a portion of dMMR tumors as sporadic.

A consistent theme is that some fraction of patients with abnormal IHC are never referred or tested. Study 5 [28], for instance, found seven patients not referred; in some cases, the advanced stage of disease or short survival overshadowed genetic concerns. Although acceptance of genetic testing was generally high once patients reached counseling, bridging that gap remains an implementation challenge in real-world settings.

## 4. Discussion

### 4.1. Summary of Evidence

The five studies examined in this review highlight how mismatch repair immunohistochemistry can serve as a front-line screening tool for detecting Lynch syndrome in CRC patients younger than 50. Despite variability in protocols—like differences in the subset of MMR proteins tested, the availability of complementary molecular markers, and the thoroughness of referral for genetic testing—each study consistently demonstrated that a subset of approximately 8% to 20% of early-onset CRC patients harbor dMMR tumors. Among these dMMR cases, the ultimate fraction confirmed as LS typically settles around 5% to 9% when universal or near-universal tumor screening and subsequent germline testing are fully implemented. This wide range partially reflects differences in the thoroughness of investigating sporadic vs. hereditary dMMR. For example, the presence of *BRAF V600E* or *MLH1* hypermethylation accounts for a large majority of sporadic dMMR with absent *MLH1*/*PMS2*. The results across studies underscore that sporadic dMMR, though less common in younger populations than in older, remains significant enough that reflex confirmatory tests are needed.

A second major finding is that partial immunohistochemistry panels—limiting testing to *MLH1* and *MSH2* alone—can miss isolated *MSH6* or *PMS2* defects. While still valuable historically, partial screening seems less ideal in contemporary practice. Indeed, diagnosing *MSH6* or *PMS2* deficiency is important since these variants, though sometimes associated with a later onset than *MLH1* or *MSH2*, still carry significant cancer risks. Another noteworthy point is the incomplete referral to genetic counseling. As the studies from The Netherlands, Japan, New Zealand, and the United States show, many patients with abnormal IHC were never tested or referred. Some were lost to follow-up or died of advanced disease. This gap can be detrimental, given the downstream implications for relatives who could benefit from cancer risk reduction strategies if LS is identified. Encouragingly, in some institutions, once patients or families reached genetic services, acceptance of germline testing was relatively high.

Third, these data underscore the relevance of tumor location (particularly right-sided), pathologic features such as tumor-infiltrating lymphocytes or medullary growth, and mention of suspicious family histories. While universal screening strategies increasingly do not rely on these features for triage, the presence of such markers can strengthen the rationale for ensuring that all younger patients receive IHC or MSI testing. As recommended by multiple guidelines, linking pathologists, surgeons, and oncologists is crucial so that these distinct features prompt timely molecular testing. Additionally, the presence of relevant family history, even if incomplete, can guide more robust genetic evaluation. The ultimate goal is to avoid missing individuals with inherited predisposition who can significantly benefit from tailored surveillance of the colon and other LS-related malignancies.

Finally, we observe an evolving shift in practice toward universal IHC testing for all CRC, not just those younger than 50, especially in high-volume academic centers. Studies have shown that 30% to 70% of LS patients might be missed if screening is restricted purely by age. Nonetheless, focusing on early-onset CRC remains a practical approach when resources are constrained. The five studies collectively reinforce the feasibility of systematically integrating IHC into routine pathology workflows for younger CRC. The benefits are clear; a meaningful fraction of new LS probands are identified, who can in turn help relatives receive appropriate genetic counseling and potentially life-saving screening. As next-generation sequencing panels become more available, future directions may streamline the distinction between sporadic dMMR and germline cases, as illustrated by some advanced approaches in these cohorts. Ultimately, bridging the gap between IHC findings, molecular confirmation, and immediate referral remains an ongoing challenge, but these studies highlight the positive clinical impact for young CRC populations

In the investigation of Lynch syndrome detection in CRC, two pivotal studies offer significant insights into the effectiveness of current screening methodologies. The study by Buchanan et al. [29] involved tumor testing for mismatch repair deficiency in two Australian CRC cohorts totaling 1639 patients diagnosed before the age of 60. This analysis revealed that 11.1% of the ACCFR cohort and 12.5% of the MCCS cohort exhibited MMR deficiency. Germline mutations were identified in 5.2% of the ACCFR group and only 0.8% in the MCCS group, highlighting a substantial variance in genetic mutation prevalence between these cohorts. A notable 41.1% and 25.2% of MMR-deficient tumors in the ACCFR and MCCS cohorts, respectively, were classified as Lynch-like, indicating an unknown etiology despite the absence of MLH1 methylation.

In a similar manner, the study by Canard et al. [30] evaluated the efficacy of LS screening in an unselected population of 1040 CRC patients undergoing surgery. Their findings indicated that 9.8% demonstrated a loss of MMR protein expression, and 9.4% exhibited microsatellite instability (MSI). Importantly, while 67.2% of cases with loss of *MLH1* expression were due to promoter methylation, 65.8% of those undergoing genetic sequencing were confirmed to have a germline mutation, underscoring the critical role of comprehensive IHC and MSI testing in identifying LS, even among those who did not meet the Bethesda criteria.

Castillejo et al. [31] focused on the prevalence of *MLH1* constitutional epimutations among CRC patients, distinguishing between unselected (*n* = 2123) and selected series (*n* = 847) who met the revised Bethesda guidelines. Their findings demonstrated a significant difference in the loss of *MLH1* expression—5.5% in the unselected series compared to 12.5% in the selected series, with constitutional epimutations only detected in the latter group (15.6% or 5 out of 32 cases). This suggests that screening for *MLH1* epimutations should be reserved for those showing clinical indications of LS per existing guidelines.

In a similar manner, the study by Chiaravalli et al. [32] explored the efficacy of universal immunohistochemical screening in a cohort of 352 consecutive CRC patients in Northern Italy. They found mismatch repair defects in 19.8% of cases, with a notable incidence of Lynch syndrome (1 in 173). The study not only reinforced the utility of IHC as an efficient method for LS screening but also demonstrated a significant patient compliance (36.8%) to genetic counseling when patients were identified through universal screening protocols.

The studies by Cavazza et al. [33] and Chika et al. [34] offer insightful perspectives into the implementation and outcomes of universal testing for Lynch syndrome in colorectal cancer patients across different geographic and healthcare settings. Cavazza et al. [33] detailed the adoption of universal mismatch repair testing in the United Kingdom, following the NICE guideline DG27, across a cohort of 198 CRC patients. Their findings showed that 11.6% (23 out of 198) of the cases were MMR-deficient, primarily in early-stage tumors, demonstrating the feasibility and efficacy of universal testing in identifying potential LS cases for personalized treatment strategies.

In a similar manner, the study by Chika et al. [34] explored the prevalence of LS and Lynch-like syndrome among a large cohort of 1234 Japanese CRC patients using a universal screening approach that included immunohistochemical analysis for MMR proteins, followed by *BRAF V600E* mutation and *MLH1* promoter methylation analysis. They identified a significantly lower prevalence of LS, with only 0.9% (11 patients) being candidates for genetic testing, of which 0.7% (9 patients) were confirmed as LS cases. This starkly contrasts with Cavazza et al.’s findings in terms of the proportion of affected individuals, highlighting potential ethnic and methodological differences in LS prevalence and detection.

### 4.2. Limitations

Our review carries several important caveats that warrant emphasis before its findings are translated into practice. First, the evidence base remains narrow—only five heterogeneous cohort studies met our inclusion criteria, most drawn from high-income, specialist centers—consequently, the pooled early-onset CRC sample of 960 patients is unlikely to capture geographic, ethnic and health-system variability, and publication bias toward well-resourced programs cannot be excluded. Second, methodological heterogeneity—partial versus complete four-antibody IHC panels, variable reflex testing for MLH1 hypermethylation or BRAF V600E, and inconsistent uptake of germline sequencing—limits direct comparison across studies and may have led to both over- and under-estimation of true LS prevalence. Third, between 20 % and 42 % of individuals with abnormal IHC were never genotyped, so the proportion of sporadic versus hereditary dMMR remains uncertain; this gap highlights real-world implementation barriers (short survival, referral delays, cost) that our aggregate data could not correct for. Fourth, none of the included studies provided long-term oncologic or psychosocial outcomes, preventing assessment of whether earlier LS detection through IHC screening translates into improved survival or cascade testing among relatives. Finally, we did not perform a formal meta-analysis because of these methodological discrepancies, which limits the statistical precision of our prevalence estimates.

## 5. Conclusions

In summary, these five studies collectively indicate that among CRC patients under age 50, approximately 8% to 20% display IHC evidence of mismatch repair deficiency, and roughly 5% to 9% ultimately prove to have Lynch syndrome on germline testing. Routine IHC-based screening, augmented by *BRAF V600E* and *MLH1* promoter methylation analysis, effectively differentiates sporadic from inherited dMMR. Our findings underscore the importance of systematically applying tumor-based screening in younger CRC patients to ensure timely referral for genetic counseling. As universal testing gains momentum worldwide, it is paramount to have robust follow-up systems for confirmatory genetic testing, enabling preventive interventions for both patients and at-risk relatives.

## Figures and Tables

**Table 1:** Basic characteristics of included studies.

Study	First Author (Year)	Country	Study Period	Number of Patients (CRC < 50 Years)	MMR Screening Method	Additional Molecular Tests (*BRAF*/*MLH1* Methylation)	Germline Testing Protocol
Study 1 [24]	Mensenkamp (2013)	Netherlands	1997–2011	25	IHC + MSI in MSI+ tumors	*BRAF* or *MLH1* tests	Sanger + IonTorrent seq
Study 2 [25]	Niessen (2006)	Netherlands	1996–2000	281	IHC for MLH1, MSH2, MSH6, etc.	*BRAF*	Denaturing gradient + MLPA
Study 3 [26]	Steinhagen (2012)	USA	2006–2010	198	IHC for 4 MMR proteins	*MLH1* methylation in *MLH1*/*PMS2* loss	Standard germline panel
Study 4 [27]	Suzuki (2017)	Japan	1996–2015	119	IHC for 4 MMR proteins	*BRAF V600E and MLH1* promoter if *MLH1*/*PMS2* loss	Sanger + MLPA if needed
Study 5 [28]	Wright (2011)	New Zealand	2001–2007	243	IHC for 2–4 proteins (evolved)	*BRAF* or Methylation	Sanger for relevant genes

**Table 2:** Prevalence of dMMR and Lynch syndrome in the five studies.

Study	dMMR Prevalence (%, 95% CI)	Confirmed LS Among Total, *n* (%, 95% CI)	Classification of dMMR Cases
Mensenkamp et al. [24]	52%	0 (0%)	Primarily biallelic somatic hits
Niessen et al. [25]	8.9%	25 (8.9%)	All dMMR → LS confirmed
Steinhagen et al. [26]	19.1%	10 (5.1%)	7 sporadic vs. 10 LS, rest VUS
Suzuki et al. [27]	8.4%	7 (5.9%)	3 sporadic, 7 LS
Wright et al. [28]	14%	12 (5.0%)	Remainder sporadic or no final test

**Table 3:** Clinicopathologic predictors of dMMR in early-onset CRC from each study. N/A: not applicable.

Study	Right-Sided Tumors in dMMR	Tumor-Infiltrating Lymphocytes (TILs) in dMMR	Mucinous/Signet Histology	Family History Mentioned
Mensenkamp et al. [24]	Not systematically reported	High TIL infiltration in many MSI + cases	Not systematically reported	N/A (unexplained MSI)
Niessen et al. [25]	60% of dMMR were right-sided	55% had TILs > moderate	20% had mucinous components	76% with first-degree hx
Steinhagen et al. [26]	53% of dMMR were right-sided	60% had TILs or Crohn-like reaction	26% mucinous or signet	55% with suspicious hx
Suzuki et al. [27]	80% of dMMR were right-sided	70% with moderate/high TILs	50% mucinous or signet	43% had first-degree hx
Wright et al. [28]	57% of dMMR were right-sided	39% had TIL mentioned in pathology	30% mucinous or signet	60% with suspicious hx

**Table 4:** Outcomes: genetic testing uptake, somatic mutation findings, and follow-up.

Study	Abnormal IHC Who Underwent Germline Testing (%)	Germline Mutation Confirmation (*n*/of Tested)	Somatic *MLH1* Hypermethylation or *BRAF V600E* (%)	Follow-Up/Registry Referral Rate
Mensenkamp et al. [24]	Not all, but 25 “unexplained” MSI used	0/25 (0%) LS found	Some tested, no universal data	High referral for further analysis
Niessen et al. [25]	100% (25/25) with dMMR tested	25/25 (100%)	Not explicitly stated	Many from known family registries
Steinhagen et al. [26]	38 dMMR; 22 (57.8%) had germline testing	10 (46% of tested); 5.1% of entire cohort	Some had *MLH1* hypermethylation (3/4 *MLH1* loss)	High counseling acceptance rate
Suzuki et al. [27]	10 dMMR; 8 tested; 80% tested	7/8 (88%); 5.9% entire cohort	2/4 *MLH1*/*PMS2* had hypermethylation, 1 had *BRAF*	Only 1 “possible LS” no final proof
Wright et al. [28]	33 dMMR; 22 (67%) tested; 10 had LS (5%)	10/22 (45%); 10 total new LS	Some tested for *BRAF*/Methylation, 7 sporadic	7 not referred, 4 died soon

## Data Availability

Not applicable.
